# Supplementary material for: Causal Relationship between Aging and Anorexia Nervosa: A White-Matter-Microstructure-Mediated Mendelian Randomization Analysis
Source: Biomedicines. 2024 Aug 16;12(8):1874. doi: 10.3390/biomedicines12081874 (PMC11351342; doi:10.3390/biomedicines12081874)
Supplement: Supplementary file 1 [file biomedicines-12-01874-s001.zip › Supplementary Figure/Figure S8/Leaveoneout plot of MR analysis for TL to ubm-b-1744.pdf]

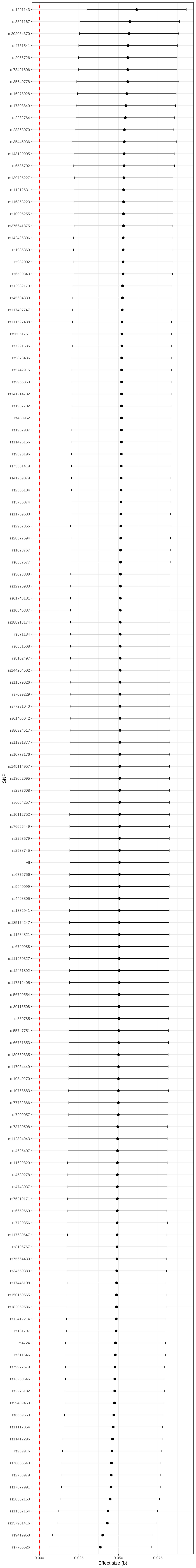
rs1291143
rs3891167
rs202034370
rs4731541
rs2056726
rs78491606
rs35640778
rs16978028
rs17803849
rs2282764
rs28363070
rs35446936
rs143190905
rs6536702
rs139795227
rs11212631
rs116863223
rs10905255
rs376641875
rs142426306
rs1985369
rs932002
rs6590343
rs12932179
rs45604339
rs117407747
rs111527438
rs56061761
rs7221585
rs9878436
rs5742915
rs9955360
rs141214782
rs1907702
rs450962
rs1957937
rs11426156
rs9398196
rs73581419
rs41269076
rs2555104
rs3785074
rs11769630
rs2967355
rs28577594
rs1023767
rs6587577
rs3093888
rs12925933
rs61748181
rs10845387
rs188918174
rs871134
rs6881568
rs8102497
rs144204502
rs11579626
rs7099229
rs77231040
rs61405042
rs80324517
rs111991877
rs10773176
rs145114957
rs13062095
rs2977608
rs6054257
rs10112752
rs76666449
rs2293579
rs2538745
All
rs6776756
rs9940099
rs4498805
rs1332941
rs185174247
rs11584821
rs6790988
rs111950327
rs12451892
rs117512405
rs56799554
rs80116508
rs869785
rs55747751
rs66731853
rs139669835
rs117034449
rs10840270
rs10768683
rs77732866
rs7209057
rs73730598
rs112394943
rs4695407
rs11699829
rs4530278
rs4743037
rs76219171
rs6659669
rs7790856
rs117630647
rs8105767
rs75664430
rs34550383
rs17445108
rs150150565
rs182059586
rs12412214
rs131797
rs4724
rs611646
rs79977579
rs13230646
rs2276182
rs59409453
rs6669563
rs111117354
rs11412296
rs939916
rs76065543
rs2763979
rs17677991
rs28502153
rs11557154
rs137901416
rs9419958
rs7705526
SNP
0.000
0.025
0.050
0.075
Effect size (b)
